# The new bridge to hernia surgery: achieving preoperative weight optimization with GLP-1 receptor agonists for abdominal wall hernia repair

**DOI:** 10.1007/s00464-025-11921-z

**Published:** 2025-07-02

**Authors:** Graham J. Spurzem, Ryan C. Broderick, Patricia Ruiz-Cota, Amanda Rocha, Edgardo Reyes, Andres Fontaine-Nicola, Agustina Altolaguirre, Hannah M. Hollandsworth, Bryan J. Sandler, Eduardo Grunvald, Garth R. Jacobsen

**Affiliations:** 1 Department of Surgery, Division of Minimally Invasive Surgery, University of California San Diego, 9300 Campus Point Dr., La Jolla, San Diego, CA 92037 USA; 2 Division of General Internal Medicine, Bariatric and Metabolic Institute, University of California San Diego, San Diego, CA USA

**Keywords:** Glucagon-like peptide 1, GLP-1, Obesity, Abdominal wall hernia, Prehabilitation

## Abstract

**Background:**

Obesity is a risk factor for complications after abdominal hernia repair. Glucagon-like-peptide-1 (GLP-1) receptor agonists are effective weight loss medications that may help patients reach weight loss goals for surgery. In this study, we examine our outcomes utilizing GLP-1 agonists for preoperative weight loss in obese patients undergoing elective hernia repair.

**Methods:**

A retrospective review identified obese patients who were prescribed GLP-1 agonists for weight loss in addition to lifestyle changes before elective hernia repair from 2021 to 2024. Patients were managed by a multidisciplinary team and asked to achieve a body mass index (BMI) ≤ 33 kg/m^2^ before surgery. Primary outcomes were preoperative mean percentage total weight loss (%TWL), mean BMI reduction, and time from GLP-1 agonist initiation to surgery. Secondary outcomes were 30-day morbidity and 30-day reoperation rates, hernia recurrence, and postoperative weight changes.

**Results:**

A total of 70 patients with ventral/incisional, umbilical, parastomal, flank, and inguinal hernias were identified. 33 patients (47.1%) underwent surgery, 24 (34.3%) remain in active follow-up, and 13 (18.6%) were lost to follow-up (no clinic visit in 1 year). For patients who underwent surgery, mean BMI on initial presentation was 37.4 ± 4.8 kg/m^2^. After initiating GLP-1 therapy, mean preoperative %TWL was 14.0 ± 6.9%. Mean preoperative BMI reduction was 5.3 ± 3.5 kg/m^2^, resulting in mean BMI at surgery of 32.0 ± 3.6 kg/m^2^. Mean time from GLP-1 agonist initiation to surgery was 8.2 ± 4.9 months. Patients available for 6-month postoperative follow-up (*N* = 7) maintained their preoperative weight loss (mean surgery BMI: 29.0 ± 2.8 kg/m^2^ vs. mean BMI at 6 months: 28.5 ± 3.4 kg/m^2^, *p* = 0.46). 30-day morbidity and reoperation rates were 9.1% and 6.1%, respectively. Hernia recurrence rate was 3.0% (*N* = 1) during a mean follow-up of 5.9 ± 12.1 months.

**Conclusion:**

GLP-1 receptor agonists can facilitate expeditious and durable preoperative weight loss for patients with obesity prior to elective abdominal wall hernia repair with low postoperative morbidity.

## Introduction

Abdominal wall hernias are common, with an estimated prevalence of 1.7% for all ages. Inguinal hernias account for approximately 75% of abdominal wall hernias, with a lifetime risk of 27% in men and 3% in women [1]. Primary ventral hernias (VH) occur in approximately 20% of adults and incisional VH can develop in up to 30% of midline abdominal incisions [2, 3]. Obesity, defined as a body mass index (BMI) greater than 30 kg/m^2^, is an independent risk factor for hernia development, recurrence, and complications after hernia repair [4–6]. Consequently, patients with obesity desiring hernia repair are often asked to meet weight loss goals before surgery in order to mitigate these risks. Lifestyle modifications, including changes in diet, physical activity, and behavioral therapy, are the cornerstone of weight loss [7]. However, pharmacologic therapy has emerged as an important adjunct to obesity management. 

Several weight loss medications have been approved by the Food and Drug Administration (FDA), and other off-label agents are currently in use [8]. Glucagon-like peptide 1 (GLP-1) receptor agonists have moved into the mainstream as pharmacologic adjuncts for weight loss, with proven safety and efficacy in patients with and without diabetes [9]. The mechanism of weight loss with GLP-1 agonists is thought, at least in part, to be due to slowed gastric emptying and increased satiety through effects on appetite centers in the brain [10]. There is a growing body of literature examining the utility of GLP-1 agonists as a tool for perioperative weight management in surgical patients, particularly in bariatrics. GLP-1 agonists can be an effective therapy for weight regain after bariatric surgery [11]. In addition, preoperative GLP-1 agonist use in bariatric patients with a BMI greater than 50 kg/m^2^ can result in significantly more weight loss compared to lifestyle interventions alone, without increased time to surgery or postoperative complication rates [12].

Little has been studied about the effectiveness of GLP-1 therapy in patients with obesity who desire hernia repair. Tran et al. showed that GLP-1 agonists can shorten the time to abdominal wall reconstruction compared to bariatric surgery for patients requiring preoperative weight loss with comparable postoperative outcomes [13]. Our group recently demonstrated that GLP-1 agonists can accelerate preoperative weight loss for obese hernia patients compared to lifestyle modifications alone without negatively impacting postoperative outcomes [14]. The goal of this study was to investigate the efficacy of GLP-1 agonists for preoperative weight loss in a larger cohort of obese hernia patients. Secondarily, we aimed to assess postoperative morbidity and examine the durability of weight loss postoperatively along with hernia recurrence rates for patients with available follow-up.

## Methods

### Study design

A retrospective, single-center review of a prospectively maintained database identified patients with obesity who were prescribed GLP-1 agonists for weight loss in addition to lifestyle changes before elective hernia repair from 2021 to 2024. Patients with a BMI greater than 33 kg/m^2^ and a ventral/incisional, umbilical, parastomal, flank, or inguinal hernia were included in the analysis. Patients with a variety of hernia types were included to evaluate the efficacy of GLP-1 therapy across a range of patient presentations and hernia morphologies. We included patients who had undergone surgery, remain in active follow-up, and patients who were lost to follow-up, defined as no clinic visit in 1 year. Patients previously prescribed GLP-1 agonists for diabetes management were excluded to isolate weight loss effects unrelated to glycemic control.

Patients were managed by a multidisciplinary weight loss team comprised surgeons, obesity medicine physicians, and registered dieticians in our comprehensive Bariatric and Metabolic Institute. Our program offers individual consultations to develop personalized weight management strategies, including diet/exercise plans and weight loss medications. Patients in the program were generally asked to achieve a BMI of 33 kg/m^2^ or less prior to hernia repair. Surgical eligibility was determined based on individual surgeon discretion, accounting for the amount of weight loss achieved, hernia morphology, and unique patient symptoms and factors. Patients prescribed GLP-1 agonists for weight loss were actively managed by a dedicated obesity medicine physician. The decision to initiate and continue these medications was based on shared decision-making discussions, the discretion of the obesity medicine physician, and insurance coverage. Insurance coverage was an important factor when deciding whether to initiate GLP-1 treatment and when choosing a particular drug. All patients who were started on a GLP-1 agonist and underwent surgery continued treatment for the duration of their preoperative course.

Demographic data included age, gender, Charlson Comorbidity Index (CCI), preoperative weight and BMI, GLP-1 medication used, other concurrently used weight loss medications, time from GLP-1 agonist initiation to surgery, and hernia type. Operative data included hernia defect size, wound classification, operative approach, and need for component separation (either Rives-Stoppa with retrorectus dissection (RR), anterior component separation with external oblique release (EOR), or posterior component separation with both RR dissection and transversus abdominis muscle release (TAR)).

### Outcomes

Primary outcomes were preoperative mean percentage total weight loss (%TWL), mean BMI reduction, and time from GLP-1 agonist initiation to surgery. Time to surgery was included as a key metric to provide context to absolute weight loss and an estimation of GLP-1 treatment duration prior surgery. Secondary outcomes were 30-day morbidity, 30-day mortality, and 30-day reoperation rates, hernia recurrence, and postoperative weight changes at 6 months and 12 months for patients with available follow-up data. Interval follow-up time and hernia recurrence were determined by either the last noted physical exam involving the abdomen or last recorded abdominal imaging in the electronic medical record.

### Statistical analysis

Categorical variables were reported as a frequency and percentage. Continuous variables were reported as a mean ± standard deviation (SD). Independent and paired sample *t*-testing were used to compare continuous variables as appropriate. For categorical variables, Fisher’s exact test was used for analyses with small samples, otherwise Pearson’s chi-square test was used. A *p*-value of < 0.05 was considered statistically significant. All statistical analysis was performed in R (Version 4.4.1; Vienna, Austria).

## Results

### Patient demographics and operative data

A total of 70 patients were identified (Table 1). Mean patient age was 57.7 ± 11.1 years and most patients (65.7%) were female. Mean BMI at initial surgery clinic visit was 39.0 ± 5.5 kg/m^2^. The most common hernia type was ventral/incisional hernia (65.7%), followed by umbilical hernia (25.7%). The majority of patients were prescribed semaglutide (74.3%) for weight loss before elective hernia repair. No patients were taking other weight loss medications concurrently with their GLP-1 agonist. Among all patients, 33 (47.1%) were deemed eligible for surgery and underwent elective hernia repair. Of the remaining patients, 24 (34.3%) remain in active weight loss follow-up. A total of 13 patients (18.6%) were lost to follow-up.

**Table 1:** Patient demographics and operative data

Patient feature	All patients (*N* = 70)
Age (years), mean (SD)	57.7 (11.1)
Female, N (%)	46 (65.7%)
CCI, mean (SD)	2.3 (1.7)
BMI (kg/m^2^) at initial clinic visit, mean (SD)	39.0 (5.5)
Hernia type, *N* (%)	
Ventral/incisional	46 (65.7%)
Umbilical	18 (25.7%)
Inguinal	3 (4.3%)
Parastomal	2 (2.9%)
Flank	1 (1.4%)
GLP-1 agonist, *N* (%)	
Semaglutide	52 (74.3%)
Tirzepatide	7 (10.0%)
Liraglutide	7 (10.0%)
Dulaglutide	4 (5.7%)
Patient status, *N* (%)	
Completed surgery	33 (47.1%)
Active follow-up	24 (34.3%)
Lost to follow-up	13 (18.6%)

*SD* standard deviation, *CCI* Charlson Comorbidity Index, *BMI* body mass index, *GLP-1* glucagon-like peptide 1

For the 13 patients lost to follow-up, mean age was 58.5 ± 11.0 years and 69.2% (*N* = 9) were female (Table 2). Mean BMI at initial surgery clinic visit was 41.3 ± 7.9 kg/m^2^. All patients presented to our surgery and/or obesity management clinics for between 1 and 3 visits in total, then failed to follow-up after attempts at rescheduling.

**Table 2:** Demographics and operative variables for patients who underwent elective hernia repair following GLP-1 therapy and patients lost to follow-up

Patient feature	Surgery patients (*N* = 33)	Lost to follow-up (*N* = 13)	*p*-value
Age (years), mean (SD)	57.3 (10.9)	58.5 (11.0)	0.72
Female, *N* (%)	19 (57.6%)	9 (69.2%)	0.47
CCI, mean (SD)	2.1 (1.6)	2.0 (1.0)	0.84
BMI (kg/m^2^) at initial clinic visit, mean (SD)	37.4 (4.8)	41.3 (7.9)	**0.04**
GLP-1 agonist, *N* (%)			0.82
Semaglutide	25 (75.8%)	11 (84.6%)	
Liraglutide	5 (15.2%)	1 (7.7%)	
Dulaglutide	2 (6.1%)	1 (7.7%)	
Tirzepatide	1 (3.0%)	0	
Hernia type, *N* (%)			0.96
Ventral/incisional	21 (63.6%)	9 (69.2%)	
Umbilical	8 (24.2%)	4 (30.8%)	
Inguinal	2 (6.1%)	0	
Flank	1 (3.0%)	0	
Parastomal	1 (3.0%)	0	
Hernia defect size (cm^2^), mean (SD)	64.3 (66.9)	60.3 (55.4)	0.85
Wound classification, *N* (%)		–	–
Class 1	29 (87.9%)		
Class 2	3 (9.1%)		
Class 3	1 (3.0%)		
Class 4	0		
Operative approach, *N* (%)		–	–
Open	18 (54.5%)		
Laparoscopic	3 (9.1%)		
Robotic	12 (36.4%)		
Component separation, *N* (%)	16 (48.5%)	–	–

*p*-values in bold indicate statistical significance

*GLP-1* glucagon-like peptide-1, *CCI* Charlson Comorbidity Index, *SD* standard deviation, *BMI* body mass index

### Preoperative weight loss and time to surgery

Demographics and operative variables for the 33 patients who underwent elective hernia repair following weight loss with GLP-1 agonists are detailed in Table 2. Mean BMI at initial surgery clinic visit for patients who underwent surgery was significantly lower than patients who were lost to follow-up (37.4 ± 4.8 kg/m^2^ vs. 41.3 ± 7.9 kg/m^2^, *p* < 0.05). Most patients who underwent surgery were prescribed semaglutide (75.8%) and had a ventral/incisional hernia (63.6%). Two patients (6.1%) reported mild nausea with GLP-1 therapy, but did not lead to treatment discontinuation.

Mean BMI at GLP-1 agonist initiation for surgical patients was 37.3 ± 4.8 kg/m^2^ and mean BMI at the time of surgery was 32.0 ± 3.6 kg/m^2^, resulting in a mean preoperative BMI reduction of 5.3 ± 3.5 kg/m^2^. These metrics were similar compared to data collected at the time of initial surgery clinic visit (Table 3). Overall, surgical patients achieved a mean preoperative %TWL of 14.0 ± 6.9% from the time of GLP-1 agonist initiation. On average, patients received 8.2 ± 4.9 months of GLP-1 therapy prior to undergoing elective hernia repair.

**Table 3:** Preoperative weight loss and time to surgery

Variable, mean (SD)	All patients (*N* = 33)
Weight (kg)	
GLP-1 agonist initiation	106.0 (18.4)
Initial surgery clinic visit	106.0 (19.0)
BMI (kg/m^2^)	
GLP-1 agonist initiation	37.3 (4.8)
Initial surgery clinic visit	37.4 (4.8)
Weight (kg) at time of surgery	91.3 (17.9)
BMI (kg/m^2^) at time of surgery	32.0 (3.6)
Preoperative %TWL	
GLP-1 agonist initiation	14.0 (6.9)
Initial surgery clinic visit	14.2 (8.4)
Preoperative BMI reduction (kg/m^2^)	
GLP-1 agonist initiation	5.3 (3.5)
Initial surgery clinic visit	5.4 (3.9)
Time to surgery (months)	
From GLP-1 agonist initiation	8.2 (4.9)
From initial surgery clinic visit	10.1 (6.7)

*GLP-1* glucagon-like peptide-1, *BMI* body mass index, *%TWL* percentage total weight loss, *SD* standard deviation

### Postoperative outcomes and weight changes

The 30-day morbidity rate was 9.1% (*N* = 3, 1 superficial incisional surgical site infection, 2 tissue flap necrosis). There were 2 reoperations within 30 days (6.1%) and no 30-day mortalities. We identified one hernia recurrence (3.0%) in a patient who developed a recurrent enterocutaneous fistula during a mean postoperative follow-up time of 5.9 ± 12.1 months.

A total of 11 patients (33.3%) elected to continue GLP-1 therapy postoperatively and 7 were available for follow-up at 6 months. These 7 patients maintained their mean day of surgery BMI at 6 months postoperatively (29.0 ± 2.8 kg/m^2^ vs. 28.5 ± 3.4 kg/m^2^, *p* = 0.46). A total of 3 patients were available for follow-up at 12 months postoperatively and also maintained their day of surgery BMI (28.1 ± 3.6 kg/m^2^ vs. 26.0 ± 5.5 kg/m^2^, *p* = 0.27).

## Discussion

Counseling patients with obesity on the importance of weight loss before elective hernia surgery has the potential to reduce postoperative complications and optimize the likelihood of a durable repair. However, weight loss with lifestyle modifications alone is often difficult, and many patients are lost to follow-up after initially being denied surgery due to high BMI [15]. In this study, we present our experience using GLP-1 agonists as a preoperative weight loss adjunct in a cohort of 70 patients with obesity and abdominal wall hernias. A total of 33 patients (47.1%) underwent elective hernia repair after preoperative weight loss at the time of this investigation and 24 patients (34.3%) remain in active weight loss follow-up. Surgical patients had relatively low postoperative morbidity (9.1%) compared to prior studies of obese hernia patients with morbidity rates as high as 35% [16].

Obesity has consistently been found to be a risk factor for both postoperative complications and recurrence after open and minimally invasive hernia repair [17]. The risks associated with obesity are likely due to multiple factors, including increased visceral fat and intra-abdominal pressure, increased abdominal wall circumference, and the pro-inflammatory state of adipose tissue, which may have adverse effects on wound healing [18, 19]. One study of 313 patients who underwent abdominal wall reconstruction for VH repair with a mean follow-up of 15.6 months found that the risk of hernia recurrence was significantly increased in patients with a higher BMI [20]. Patients with a BMI of 30–34.9 kg/m^2^ had a recurrence rate of 29.8%, compared to 8.3% and 12.5% for patients with BMIs of 15–24.9 kg/m^2^ and 25–29.9 kg/m^2^, respectively. Some investigations have attempted to identify a BMI threshold for a significant increase in complications and recurrence [21, 22]. Other authors advocate for a more individualized approach to preoperative weight loss, especially considering the time required to achieve significant weight loss and the risk of a surgical emergency developing [23]. Nevertheless, hernia specific studies have shown that meaningful preoperative weight loss is challenging. In a study of 191 hernia patients who were offered a free preoperative weight loss program, 55% of enrolled patients were lost to follow-up and only 10% of enrolled patients met the prescribed weight loss goal and underwent hernia repair [24]. In a randomized trial of obese patients with ventral hernias analyzing the effect of a prehabilitation program, only 20% of patients were able to meet the 7% weight loss benchmark [25]. Bariatric surgery has been proposed as a bridge therapy to hernia repair, but this approach may ultimately further delay the time to hernia surgery and many patients may face barriers to accessing bariatric care [26, 27]. GLP-1 agonists present a promising opportunity to help patients achieve preoperative weight loss goals and may help hernia prehabilitation programs to increase patient retention.

In our cohort, 47.1% of patients who were prescribed GLP-1 agonists were able to achieve substantial preoperative weight loss and undergo hernia surgery. On average, these patients had a mean total weight loss of 14% from the time of starting GLP-1 therapy to the day of surgery. Our weight loss outcomes and the time needed to achieve them are comparable to larger prospective and retrospective studies on GLP-1 agonists. The Semaglutide Treatment Effect in People with Obesity (STEP) randomized trials found that patients receiving semaglutide 2.4 mg lost an average of 6% of their total weight by week 12 and 12% of their total weight by week 28 [28]. In a retrospective cohort study of 175 patients with obesity prescribed semaglutide for weight loss, the mean weight loss after 6 months was 12.3 kg, equivalent to a mean total weight loss of 10.9% [29]. In addition, 54.9% of patients in the study achieved a total weight loss of 10% or more at 6 months. While only 7 patients in our cohort elected to continue GLP-1 therapy postoperatively and were available for follow-up at 6 months, these patients effectively maintained their preoperative weight loss. Large-scale randomized trials have shown that the weight loss trajectory with semaglutide occurs over a period of 65 weeks and is sustained for up to 4 years [30]. Although the impact of postoperative GLP-1 therapy on long-term hernia-related outcomes is unknown, it’s feasible that maintaining weight loss after surgery with GLP-1 agonists may help to reduce the likelihood of postoperative complications and hernia recurrence. Prospective studies with long-term follow-up are needed to compare the weight loss efficacy of GLP-1 agonists with and without lifestyle modifications in hernia patients to investigate this question. A quantifiable benefit of these medications with regard to hernia complications may support broader use of GLP-1 agonists for hernia prehabilitation.

Nearly 1 in 5 patients in this study were lost to follow-up despite being referred to obesity management and advised to return to our surgery clinic for a weight loss check. Based on our retrospective review, the reasons for this are unclear. It’s likely that many patients became discouraged and unmotivated when preoperative weight loss was recommended instead of scheduling surgery on the first visit. Some patients also may have not responded to GLP-1 therapy or experienced significant side effects and elected to discontinue treatment. GLP-1 agonists can have a variable weight loss effect that is not completely understood. Proposed mechanisms for this variability include polymorphisms in the GLP-1 receptor and responses also may vary based on age, gender, BMI, and comorbidities [31–35]. We did find that patients who were lost to follow-up had a significantly higher average BMI on initial clinic visit compared to patients who went on to lose weight and have surgery. It’s unclear if these patients viewed the recommended weight loss as insurmountable and sought elective hernia repair at another institution or chose to forego surgery altogether. We also had limited insight into patient understanding of the relationship between obesity and postoperative outcomes. It’s possible that patients who continued to take GLP-1 therapy and follow-up for obesity management were a self-selecting group motivated to minimize the risk of postoperative complications. Although our study and the literature more broadly suggest that preoperative weight loss efforts can be beneficial for some patients, the criteria for selecting ideal candidates for preoperative GLP-1 therapy remain unclear. The optimal strategy for recruiting and retaining patients with higher BMIs and other potential risk factors for not achieving adequate preoperative weight loss also needs further study.

The availability of GLP-1 agonists in terms of insurance coverage is an important consideration as well. Given the high cost of GLP-1 agonists and their rising popularity, insurers have employed strategies to control drug costs. For example, many insurance plans in the Affordable Care Act (ACA) marketplaces manage the costs associated with GLP-1 agonists by only covering them for diabetes and not for weight management [36]. The 2024 Kaiser Family Foundation (KFF) Health Benefits Survey found that approximately 20% of employers cover GLP-1 agonists for weight loss and 33% of these firms say that covering GLP-1 agonists for weight loss will have a “significant impact” on their prescription drug spending [37]. Telehealth companies have emerged offering compounded semaglutide at a fraction of the cost of FDA-approved brand-name medications in an effort to capture a portion of the market share. However, compounded GLP-1 agonists are not FDA-approved and there have been numerous reports of adverse events associated with these drugs [38]. Brand-name drugs are also associated with side effects, including nausea, vomiting, diarrhea, and even severe hypoglycemia and pancreatitis in large clinical trials [39]. The variable insurance landscape and potential for adverse effects from multiple drug sources highlight the importance of collaboration between surgeons and obesity medicine specialists when managing patients on GLP-1 agonists. In our experience, this collaboration has been essential for navigating weight loss medication options and preoperative weight management.

There are several limitations to this retrospective single-center study, including its limited sample size, follow-up time, and low rates of postoperative follow-up. A prospective trial design may have improved our follow-up rates and/or allowed us to identify the reasons patients decided not to follow-up. In particular, our mean follow-up time of 5.9 months is too short to robustly evaluate hernia recurrence, and we plan to continue collecting long-term data on this cohort. Given that access to GLP-1 agonists is highly dependent on insurance coverage and financial resources, there may be socioeconomic factors biasing the composition of our cohort. We also recognize that including various hernia types introduces complexity into our analysis, however, we believe our heterogeneous patient population supports the utility of GLP-1 therapy in a variety of hernias and enhances the generalizability of our findings. Further studies are warranted to evaluate the safety and efficacy of GLP-1 agonists for the perioperative weight management of surgical patients in other disciplines. The utility of postoperative GLP-1 agonist therapy and the long-term impact of these medications on hernia-related outcomes, including hernia recurrence, also warrants investigation.

## Conclusion

GLP-1 receptor agonists can facilitate expeditious and durable preoperative weight loss for patients with obesity prior to elective abdominal wall hernia repair with low postoperative morbidity.
